# Targeting SIRT1 via Exosomes Derived From Oleuropein‐Treated Cells: A Novel Approach to Rejuvenation Skin Through miRNA Modulation

**DOI:** 10.1002/fsn3.70964

**Published:** 2025-09-14

**Authors:** Naeimeh Safavizadeh, Zahra Noormohammadi, Mohammad Zaefizadeh, Kazem Nejati Koshki

**Affiliations:** ^1^ Department of Biology, Science and Research Branch Islamic Azad University Tehran Iran; ^2^ Department of Biology Ardabil Branch, Islamic Azad University Ardabil Iran; ^3^ Pharmaceutical Sciences Research Center Ardabil University of Medical Sciences Ardabil Iran

**Keywords:** exosome, miRNAs, oleuropein, *SIRT1*

## Abstract

*Sirtuin1* (*SIRT1*) plays an important role in skin aging by regulating cellular processes such as oxidative stress response, inflammation modulation, *Collagen* and *Elastin* synthesis. This study aims to examine oleuropein's (OLE) effect on *SIRT1* gene expression and to analyze *SIRT1*‐related miRNAs in exosomes produced from Mesenchymal Stem cells (MSC) and Human Fetal Foreskin Fibroblast 2 (HFFF2) cells, along with these treated exosomes' impact on *SIRT1* gene expression and the studied miRNAs in HFFF2 cells to decrease skin aging. A nontoxic concentration (400 μg/mL) of OLE was applied to the MSCs and HFFF2 cells. Then, Gradient Ultracentrifugation extracted their exosomes; cell‐derived exosomes were confirmed by DLS assay and western Blot. Exosomes were applied at 50 μg/mL (exosome protein concentration) to HFFF2 cells. The expression of *SIRT1* gene and related miRNAs relative to the control group were examined using qRT‐PCR. This analysis was conducted on cells OLE‐treated for *SIRT1*, on exosomes treatment with OLE for miRNAs, and on HFFF2 cells treated with cell‐derived exosomes for both *SIRT1* and miRNAs. *SIRT1* expression was upregulated (*p* ≤ 0.05) in both OLE and cell‐derived exosomes. Also, *hsa‐miR‐29c‐3p* and *hsa‐miR‐9‐5p* were downregulated (*p* ≤ 0.05), whereas *hsa‐miR‐155‐5p* was upregulated (*p* ≤ 0.05) in exosomes OLE‐treated and in HFFF2 cells treated with these exosomes. This study introduces a novel approach to skin rejuvenation by using manipulated exosomes OLE‐treated, which enhance *SIRT1* expression and suppress related miRNAs. This method potentially offers a more effective and less immunogenic alternative to direct OLE application due to the exosomes' ability to penetrate cells.

## Graphical abstract

Treatment with OLE and its derived exosomes significantly upregulated SIRT1 expression while modulating specific miRNAs (downregulating hsa‐miR‐29c‐3p and hsa‐miR‐9‐5p, and upregulating hsa‐miR‐155‐5p), with exosome‐mediated delivery potentially offering a more effective approach than direct OLE treatment due to enhanced cell penetration and reduced immune activation.

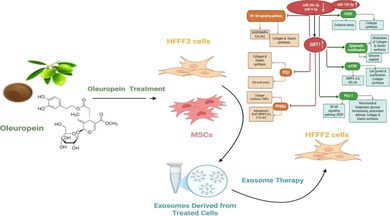

## 1 Introduction

Skin is an effective barrier against a range of external infections and environmental harm because it is an organ in direct contact with the outside world (Rawal et al. 2023). As time passes, the skin undergoes natural aging processes influenced by a variety of factors, both genetic and environmental. Nowadays, there is a growing trend of including novel substances or medications for combating skin aging in research and making them available for purchase (Cuffaro et al. 2023; Hernandez et al. 2021; Sharma et al. 2022; Yang et al. 2024).

Research indicates that olive is one of the substances that contribute to skin rejuvenation; the anti‐photoaging action and other skin benefits of olive extracts, which are botanical components of many nutricosmetic and cosmetic treatments, are believed to be due, in part, to OLE (Machała et al. 2022). OLE is a phenolic phytochemical and an antioxidant that enhances olive's biological activity in vitro, including antioxidant, antibacterial, anticarcinogenic, and anti‐glycemic properties (Wanitphakdeedecha et al. 2020). Clinical trials have shown that emulsions and creams containing OLE have significant anti‐aging and photoprotective properties. These preparations have been found to decrease erythema, dehydration, blood flow, and trans‐epidermal water loss caused by UVB irradiation (Klieser et al. 2019; Yang et al. 2022). OLE has the potential to reduce the aging process of the skin since it can control the activity of *SIRT1*. This highly conserved NAD^+^‐dependent deacetylase is crucial to control a variety of cellular processes (Abdelmohsen et al. 2007). Research has shown that *SIRT1* is essential for metabolic regulation and adaptability (Csekes and Račková 2021). Furthermore, oxidative stress reduced *SIRT1*'s mRNA and protein levels in HDFs (Csekes and Račková 2021). Also, *SIRT1* has been implicated in the modulation of inflammatory pathways and the promotion of antioxidant defenses, both of which are critical in counteracting the deleterious effects of skin aging. Studies have demonstrated that OLE can activate *SIRT1*, leading to a cascade of downstream effects that contribute to the preservation of skin health and the attenuation of age‐related skin changes (Kose et al. 2022; Shao et al. 2020). Current anti‐aging strategies frequently use topical retinoid and antioxidants to decrease these effects. However, these traditional approaches often face limitations involving skin irritation, photosensitivity, and restricted cellular penetration, thereby limiting their overall effectiveness (Narsa et al. 2024).

A novel approach in dermatology is the utilization of exosomes. Exosomes, a form of extracellular vesicle, are microscopic particles produced by cells that have important roles in the regulation of both normal and abnormal skin processes (Thakur et al. 2023). Exosomes help cells communicate by transporting miRNAs, mRNAs, DNA, lipids, metabolites, and cell‐surface proteins (Kalluri and LeBleu 2020). Exosomes supply significant advantages, including high stability, non‐immune rejection, and the ability to directly stimulate target cells (Baptista et al. 2021). Recent advancements in regenerative medicine have indicate the potential of exosome‐based therapies as a novel and more effective alternative. Exosomes, nanoscale vesicles secreted by cells, provide enhanced cellular penetration and reduced immunogenicity compared to conventional methods (Moghassemi et al. 2024).

In particular, the miRNAs found in exosomes are involved in controlling different cellular responses by binding to the 3′‐untranslated region (Zeng et al. 2020). Their primary role is to regulate gene expression at the posttranscriptional level (Qian et al. 2024). Manipulating miRNA content in exosomes will be essential for advancing future therapies. Thus, the extraction, purification, and modification of exosomal cargo are essential to enhance the efficacy of miRNA‐based therapy and maximize its production (Olsson et al. 2019). Recent studies indicate that exosomal miRNAs derived from MSCs are crucial in skin rejuvenation. They achieve this by targeting several genes and regulating different biological processes, for instance inflammatory responses, cell migration, proliferation, and apoptosis (Zheng et al. 2024).

In this study, specific miRNAs were investigated that target the SIRT1 gene, including *miR‐29c‐3p* (Wang et al. 2021), *miR‐9‐5p* (Wu et al. 2023), and *miR‐155‐5p* (Ke et al. 2023; Zeng et al. 2023). These miRNAs may significantly influence the aging process of the skin by targeting the *SIRT1* gene and its associated signaling pathway. The research was studied the effects of OLE on *SIRT1* gene expression and the production of manipulated exosome content in MSCs and HFFF2 cells. Additionally, it was examined how these manipulated exosome content affect *SIRT1* gene expression and the related miRNAs in HFFF2 cells.

## 2 Materials and Methods

### 2.1 Cell Culture and Cell Viability

Human mesenchymal stem cells (MSCs) were cultured following the methodology proposed by Maleki et al. (2014). Also, the human forehead fibroblast cell line (HFFF2) was bought from the Pasteur Institute, Tehran, Iran, and cultured in fibroblast medium LG‐DMEM (Sigma Aldrich; USA) at 37°C in the presence of 5% CO2 at 96% humidity atmosphere, as previously described (Safavizadeh et al. 2025).

The cell viabilities of MSCs and HFFF2 cell lines treated with OLE (Sigma Aldrich, USA, 32619‐42‐4) were measured by the MTT (2‐(4,5‐dimethythiazol‐2‐yl)‐2,5‐diphenyltetrazolium bromide) assay, following established protocols (Safavizadeh et al. 2025). Briefly, MSCs and HFFF2 cells were seeded in 96‐well plates at 6 × 10^3^ cells per well and permitted to attach for 24 h. OLE (0‐2000 μg/mL) was incubated with the cells for 24 and 48 h, followed by adding the MTT solution (Sigma Aldrich, USA) in each well and incubated for 4 h. Next to incubation for 4 h, the content of the wells was eliminated, and dimethyl sulfoxide (DMSO) was added to the wells (the assay was repeated three times). The optical density of each well was recorded at a wavelength of 570 nm using a plate reader (Awareness Microwell Plate Reader Chromate 4300, USA).

### 2.2 MSCs and HFFF2 Cells Treatment With OLE

The MSCs and HFFF2 cell lines were cultured, as previously explained (Safavizadeh et al. 2025) for 24 h before the treatment in flasks, followed by treatment with nontoxic concentration OLE (IC_25_ ≃ 400 μg/mL) for an additional 24 h the next day.

### 2.3 Isolation and Confirmation of Exosomes

#### 2.3.1 Isolation of Exosomes

After 24‐h treatment with OLE, cells were collected and transferred the culture cell supernatants into centrifuge microtubes. Following established protocols (Safavizadeh et al. 2025), exosomes were isolated from cell culture through ultracentrifugation, utilizing optimized protocols provided by Thermo Fisher Scientific, USA. In summary, the supernatants were centrifuged in an order: first at 300×*g* for 10 min, followed by 2000×*g* for another 10 min, both conducted at 4°C and finally at 10000×*g* for 30 min, also at 4°C. Next, the supernatants were being ultracentrifuged at 100,000×*g* for 90 min at a temperature of 4°C. Subsequently, the pellets were resuspended in phosphate‐buffered saline (PBS) and ultracentrifugated at 100,000×*g* for an additional 90 min at 4°C. The pellets were finally resuspended in 500 μL of PBS and then stored at −80°C for storage.

#### 2.3.2 Exosomes Confirmation

Exosome conformation was employed by Dynamic Light Scattering (DLS) and Western Blot. As previously described (Safavizadeh et al. 2025), the sizes of the isolated MSC‐derived exosomes and HFFF2‐derived exosomes were measured by DLS (Horiba‐SZ‐100Z, Japan), applying the subsequent parameters: Viscosity: 0.894 mPa·s and Refractive Index: 1.330.

Also, 500 μL of isolated MSC‐derived exosomes and HFFF2‐derived exosomes, resuspended in PBS, were lysed using lysis buffer. After centrifugation at 12,000*g* for 10 min at 4°C, we extracted the protein‐containing supernatant and stored it in a freezer at −20°C. We quantified the protein concentrations using the Bradford assay. We isolated the quantified proteins using a 10% polyacrylamide gel and electro‐transferred them to polyvinylidene fluoride (PVDF, Thermo Scientific, USA) membranes. The membranes were blocked with 5% BSA (Roche, Germany) for two hours. After that, they were incubated for 18 h with primary antibodies that targeted CD63 (Santa Cruz, USA, #sc‐5275), CD9 (Santa Cruz, USA, #sc‐18869), and β‐actin (Santa Cruz, USA, #sc‐47778). After washing, we incubated the membranes for 1 h with secondary anti‐rabbit (Santa Cruz, USA, #sc‐2357). We applied enhanced chemiluminescence (ECL) for band detection and used ImageJ software for analysis. This study characterized exosome preparations through immunoblotting for CD63 and CD9, which are established as accepted exosomal surface markers. These proteins are frequently utilized to verify the presence of exosomes, owing to their concentration in the exosomal membrane and their recognized functions in exosome biogenesis and trafficking (Aliakbari et al. 2024; Khushman et al. 2017). It is essential to recognize that dependence exclusively on CD63 and CD9 presents certain limitations. Recent guidelines and studies advocate for the inclusion of more types of exosomal markers, such as CD81, alongside cytosolic proteins like TSG101 and Alix, to more thoroughly validate the identity and purity of exosomes (Aliakbari et al. 2024; Zhang et al. 2024).

### 2.4 Cell Line Treatment With Isolated Exosomes

As previously indicated (Safavizadeh et al. 2025), before treatment, HFFF2 cell lines were cultured in culture flasks for 24 h then they were treated with MSC‐derived exosomes and HFFF2‐derived exosomes 50 μg/mL (exosomes' protein concentration synchronizes) for an additional 24 h.

### 2.5 In Silico Analysis of miRNAs Related to *SIRT1*

In silico analysis was utilized to confirm the selected miRNAs related to *SIRT1* by several algorithms, including TargetScan, miRDB, miRTarBase, miRNet, miRbase, BiBiServ2‐RNAhybrid, and the Ensembl genome browser 112 databases as previously explained (Safavizadeh et al. 2025). These algorithms predict that *SIRT1* can be a target gene for miRNAs based on several parameters, including the presence or absence of complementary miRNAs binding sites in the 3′‐UTR of *SIRT1* mRNA and the minimum binding energies.

### 2.6 RT‐qPCR Analyses of *SIRT1* Gene and Related miRNAs

After 24 h, following established protocols (Safavizadeh et al. 2025), total RNA was isolated utilizing the EX6101‐RNX Plus Solution (SinaClone, Iran) from OLE‐treated MSC and HFFF2 cells (IC_25_ ≃ 400 μg/mL), HFFF2 cells treated with exosomes derived from OLE‐treated cells (exosomes' protein concentration ≃ 50 μg/mL) and control groups, following the manufacturer's instructions. On the other hand, the RNeasy Mini Kit (Qiagen, Germany) was used to isolate the miRNAs from exosomes derived from OLE‐treated MSC, HFFF2 cells and also HFFF2 cells treated with exosomes derived from OLE‐treated cells, and control groups according to the manufacturer's instructions. The genomic DNA‐free RNA was analyzed using a spectrophotometer (Thermo Fisher Scientific, USA) at an absorption wavelength of 260/280 nm. Subsequently, total isolated RNAs were used to synthesize complementary DNA (cDNA) utilizing the SinaClon First Strand cDNA Synthesis Kit (SinaClone, Iran), following the manufacturer's protocol. Additionally, cDNA from isolated miRNAs was synthesized using specific miRNA Stem‐Loop primers and the SinaClon First Strand cDNA Synthesis Kit (SinaClone, Iran).

*SIRT1* relative gene expression was measured by q RT‐PCR, utilizing the gene‐specific primers shown in Table 1. The relative expression of genes was examined at two steps: first step: MSCs, HFFF2 cells treated with OLE, and control groups; second step: HFFF2 cells treated with MSC‐derived exosomes OLE‐treated, HFFF2‐derived exosomes OLE‐treated, and control groups. Also, expression of the *miR‐29c‐3p*, *miR‐9‐5p*, and *miR‐155‐5p* miRNAs were examined utilizing the miRNAs‐specific primers presented in Table 1. The expression of these miRNAs was analyzed at two steps: first step: MSC‐derived exosomes, HFFF2‐derived exosomes treated with OLE, and control groups; second step: HFFF2 cell lines treated with MSC‐derived exosomes OLE‐treated, HFFF2‐derived exosomes OLE‐treated, and control groups. The gene amplification was performed using the Sina SYBR Blue HS‐qPCR kit (SinaClone, Iran) by the manufacturer's protocol. Relative analysis at the mRNA level was employed with two replicates using the Pfaffian method with *e* ^−ΔΔct^ compared with the *GAPDH* and *U6* housekeeping genes (Table 1).

**TABLE 1:** Gene and miRNAs primer sequences used for q RT‐PCR.

Gene and miRNA	Primer sequence (5′–3′)
SIRT1	Forward: 5′‐CTGGACAATTCCAGCCATCT‐3′ Reverse: 5′‐GCACCTAGGACATCGAGGAA‐3′
GAPDH	Forward: 5′‐TCCCTGAGCTGAACGGGAAG‐3′ Reverse: 5′‐GGAGGAGTGGGTGTCGCTGT‐3′
miR‐29c‐3p	Forward: 5′‐GTTGGGTAGCACCATTTGAAAT‐3′ Reverse: 5′‐GTGCAGGGTCCGAGGT‐3′
miR‐9‐5p	Forward: 5′‐GTTGGTCTTTGGTTATCTAGCT‐3′ Reverse: 5′‐GTGCAGGGTCCGAGGT‐3′
miR‐155‐5p	Forward: 5′‐GGGGTTAATGCTAATCGTGATA‐3′ Reverse: 5′‐GTGCAGGGTCCGAGGT‐3′
U6	Forward: 5′‐GCTTCGGCAGCACATATACTAAAAT‐3′ Reverse: 5′‐CGCTTCACGAATTTGCGTGTCAT‐3′

### 2.7 Statistical Analysis

Relative gene expression data was used in analysis of variance (ANOVA) at *p* ≤ 0.05. Prismv10.3.1.509 software (GraphPad Software Inc., La Jolla, CA) was used for statistical data processing and generating diagrams.

## 3 Results

### 3.1 Viability Percentage

**FIGURE 1:**
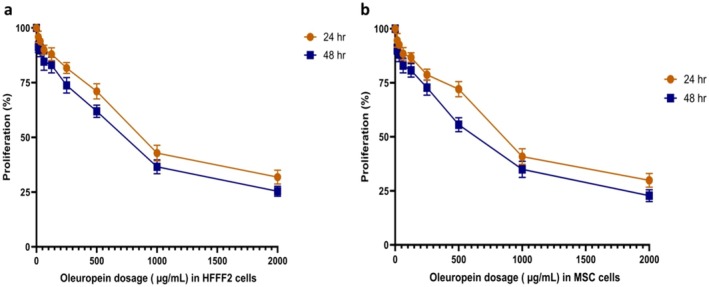
Cell viability. (a) HFFF2 cell line treated with OLE in MTT assay; (b) MSCs cell line treated with OLE in MTT assay.

We analyzed how different concentrations of OLE affected the viability of the HFFF2 cell line and MSCs (Figure 1). The IC_50_ was determined to be 855.69 μg/mL for the HFFF2 cell line and 814.05 μg/mL for MSCs. The IC_25_ (approximately 400 μg/mL) was employed for cell treatment as an appropriate concentration, IC_25_ was used to decrease damage and protect the safety of the cells. Study indicates that OLE exhibits cell inhibition and toxicity at considerably higher concentrations, with an IC_50_ concentration approximately equal to 800 μg/mL.

### 3.2 Exosomes Confirmation

**FIGURE 2:**
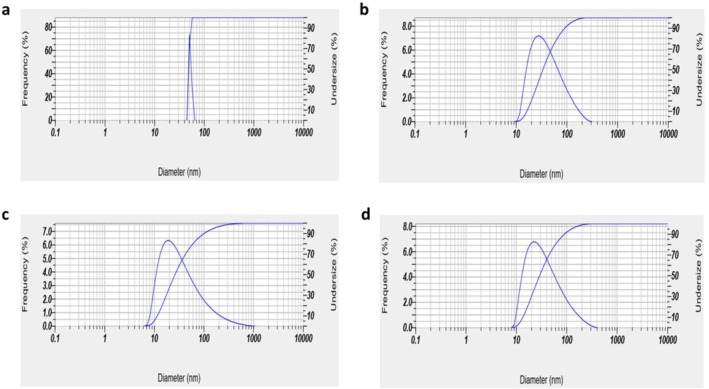
DLS to characterize the size of exosomes. (a) Exosomes derived from MSCs treated with OLE, (b) Exosomes derived from MSCs control group, (c) Exosomes derived from HFFF2 cell line treated with OLE, (d) Exosomes derived from HFFF2 cell line control group.

Dynamic Light Scattering (DLS): The sizes of exosomes isolated from MSCs treated with OLE and the control group were 49.2 ± 1.64 and 45.4 ± 5.9 nm, respectively. The HFFF2 cell line's exosomes were 47.3 ± 8.01 nm in size from the OLE‐treated group and 42.8 ± 6.3 nm in size from the control group, as shown in DLS analyses (Figure 2a,b). The small size of the exosomes resulted from using a centrifuge that operated at nearly 100,000×*g*.

**FIGURE 3:**
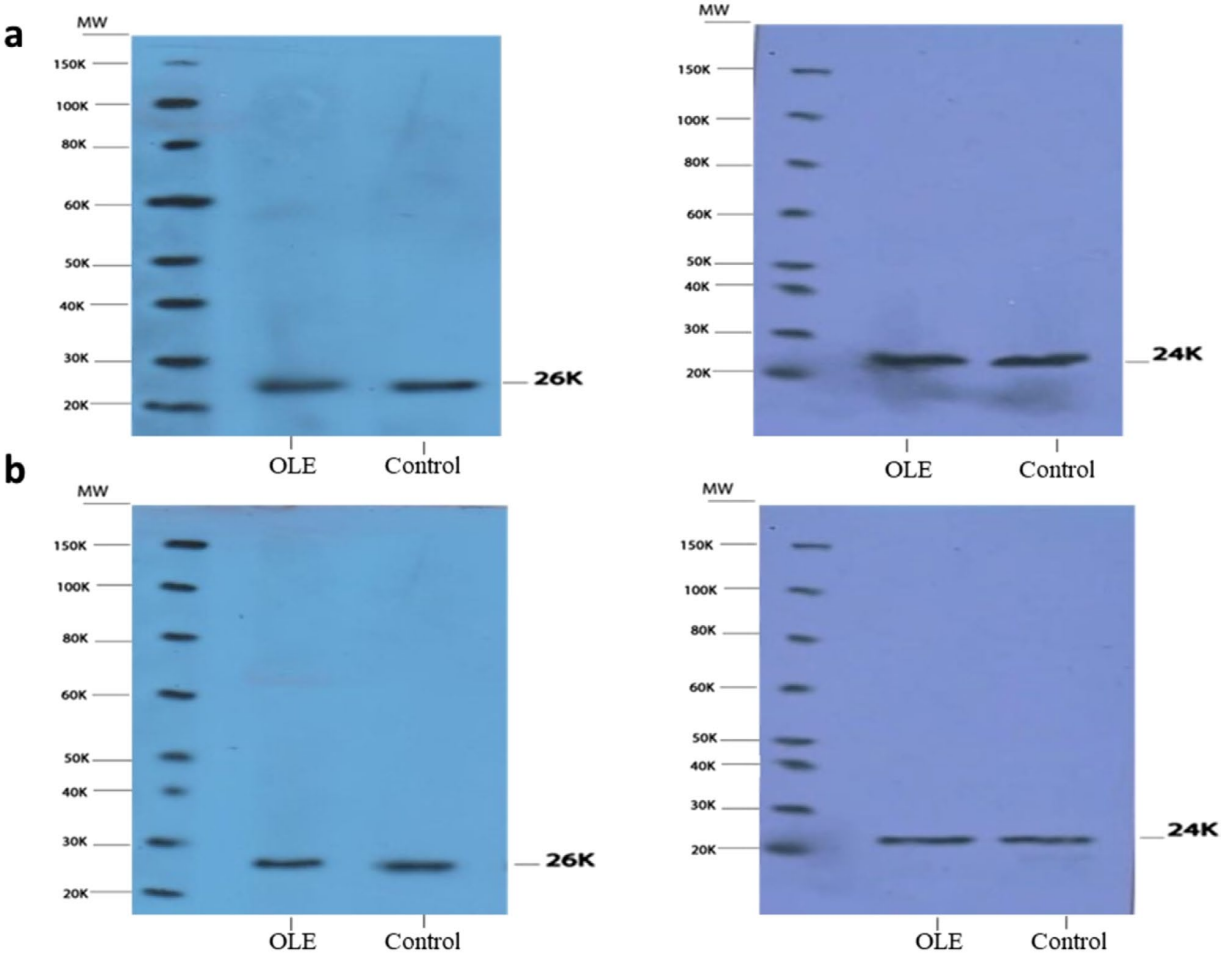
Western blot. (a) CD63 (26 kDa) and CD9 (24 kDa) bands in HFFF2 cells; (b) CD63 (26) and CD9 (24 kDa) bands in MSCs cells.

The result of western blot showed protein bands at 26 and 24 kDa (Figure 3a,b), which are the same size as the exosome membrane proteins CD63 and CD9, respectively. The exosomes' special marker membrane proteins CD63 and CD9 were detected in the MSCs and HFFF2 cell lines treated with both the OLE and control groups.

### 3.3 In Silico Analysis of miRNAs Related to *SIRT1*

In silico analysis was conducted to evaluate and confirm the selection of miRNAs related to *SIRT1* (Table 2). The miRNAs including *miR‐29c‐3p*, *miR‐9‐5p*, and *miR‐155‐5p* were chosen for investigation due to their potential roles in regulating SIRT1 and the extracellular matrix (ECM) remodeling pathways, which are crucial for skin aging.

**TABLE 2:** The list of selected miRNAs related to *SIRT1*.

Target gene	miRNAs	References
SIRT1 Sirtuin 1	miR‐29c‐3p miR‐9‐5p miR‐155‐5p	(Ke et al. 2023; Wang et al. 2021; Wu et al. 2023; Zeng et al. 2023)

### 3.4 The *SIRT1* Gene and Related miRNA Expressions

#### 3.4.1 The *SIRT1* Gene Expression OLE‐Treated

**FIGURE 4:**
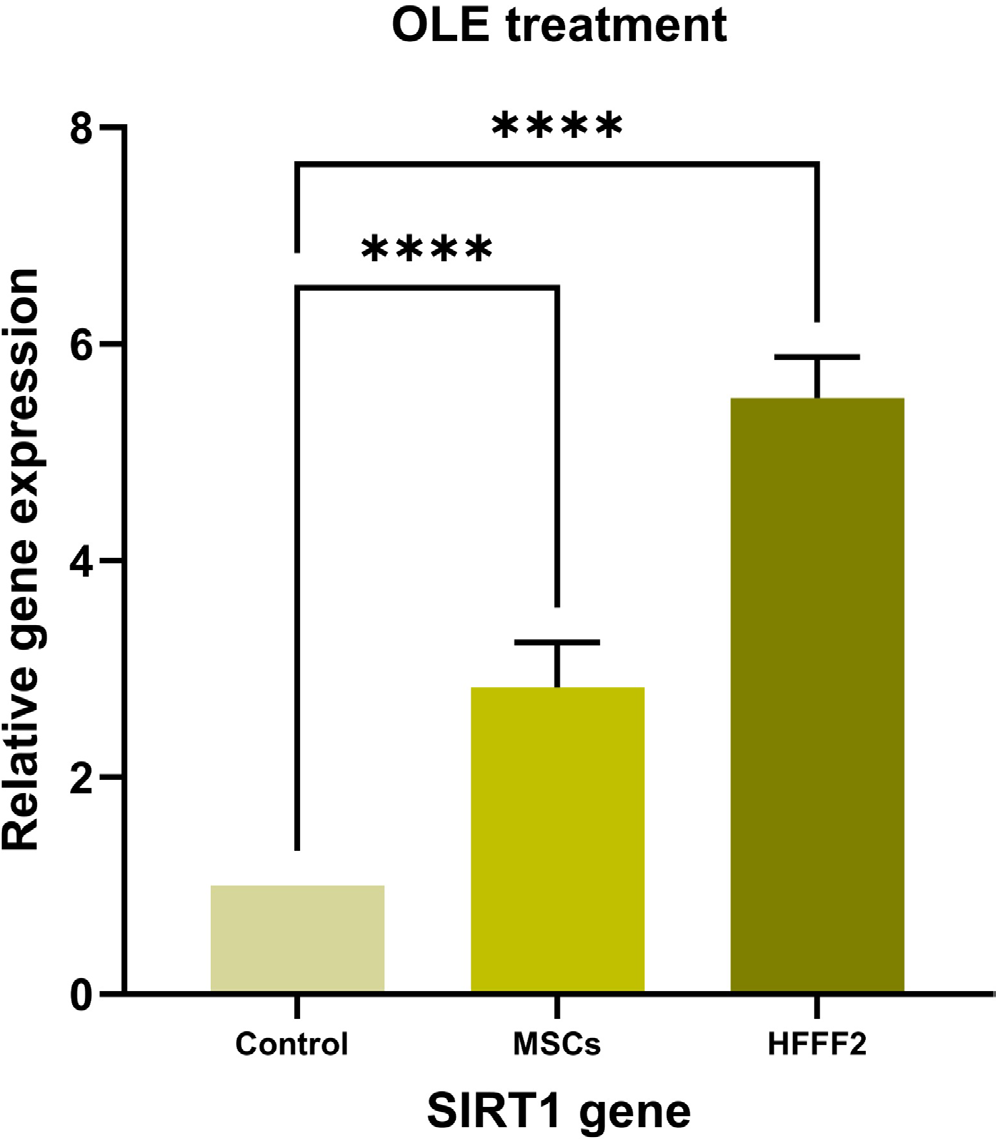
Relative expression of SIRT1 gene in MSCs and HFFF2 cells under OLE treatment and the control, (ns) nonsignificant, (*) *p* ≤ 0.05, (**) *p* ≤ 0.01, (*** or more) *p* ≤ 0.001.

The result of ANOVA showed that the relative expression of the *SIRT1* gene in MSCs and HFFF2 cells was different between the OLE treatment and control (*p* ≤ 0.05). Consequently, the treatment has modified the expression of *SIRT1* in the both of cells (Figure 4). OLE treatment significantly increased the expression of *SIRT1* in the MSCs (2.83: fold change) and HFFF2 cells (5.5) compared to the control group (Figure 4).

#### 3.4.2 The *SIRT1* Gene Expression Treated With Manipulated Exosomes

**FIGURE 5:**
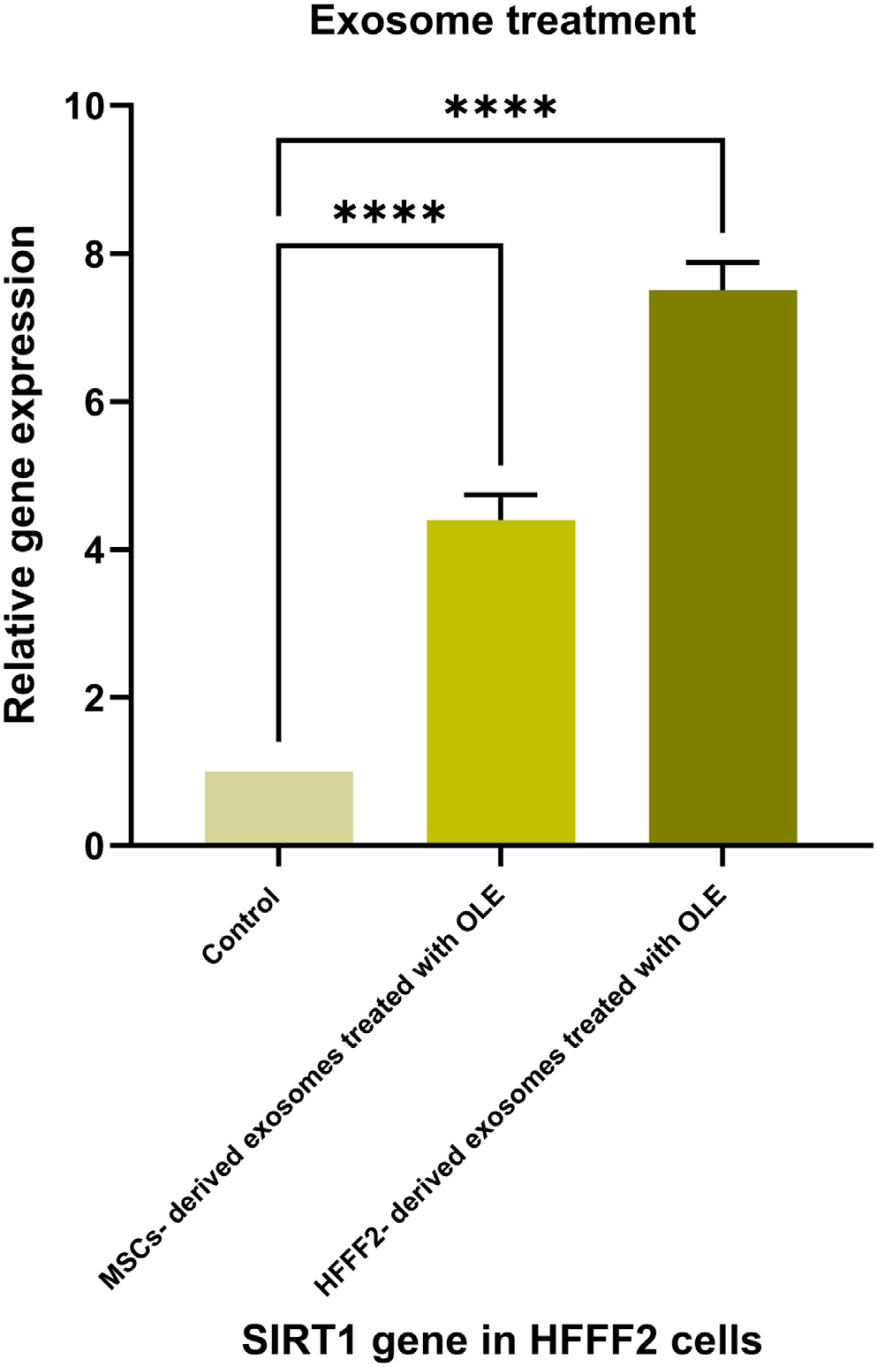
Relative expression of SIRT1 gene treated with manipulated (OLE‐treated) exosomes in HFFF2 cell line, (ns) nonsignificant, (*) *p* ≤ 0.05, (**) *p* ≤ 0.01, (*** or more) *p* ≤ 0.001.

The analysis of variance (ANOVA) for the relative expression of the *SIRT1* gene under the treatment of MSCs‐ derived exosomes OLE‐treated (4.4) and HFFF2‐ derived exosomes OLE‐treated (7.5) in the HFFF2 cell line revealed a significant difference between the treatments (*p* ≤ 0.05) (Figure 5).

#### 3.4.3 The miRNAs Expression in Exosomes Derived From OLE‐Treated Cells

**FIGURE 6:**
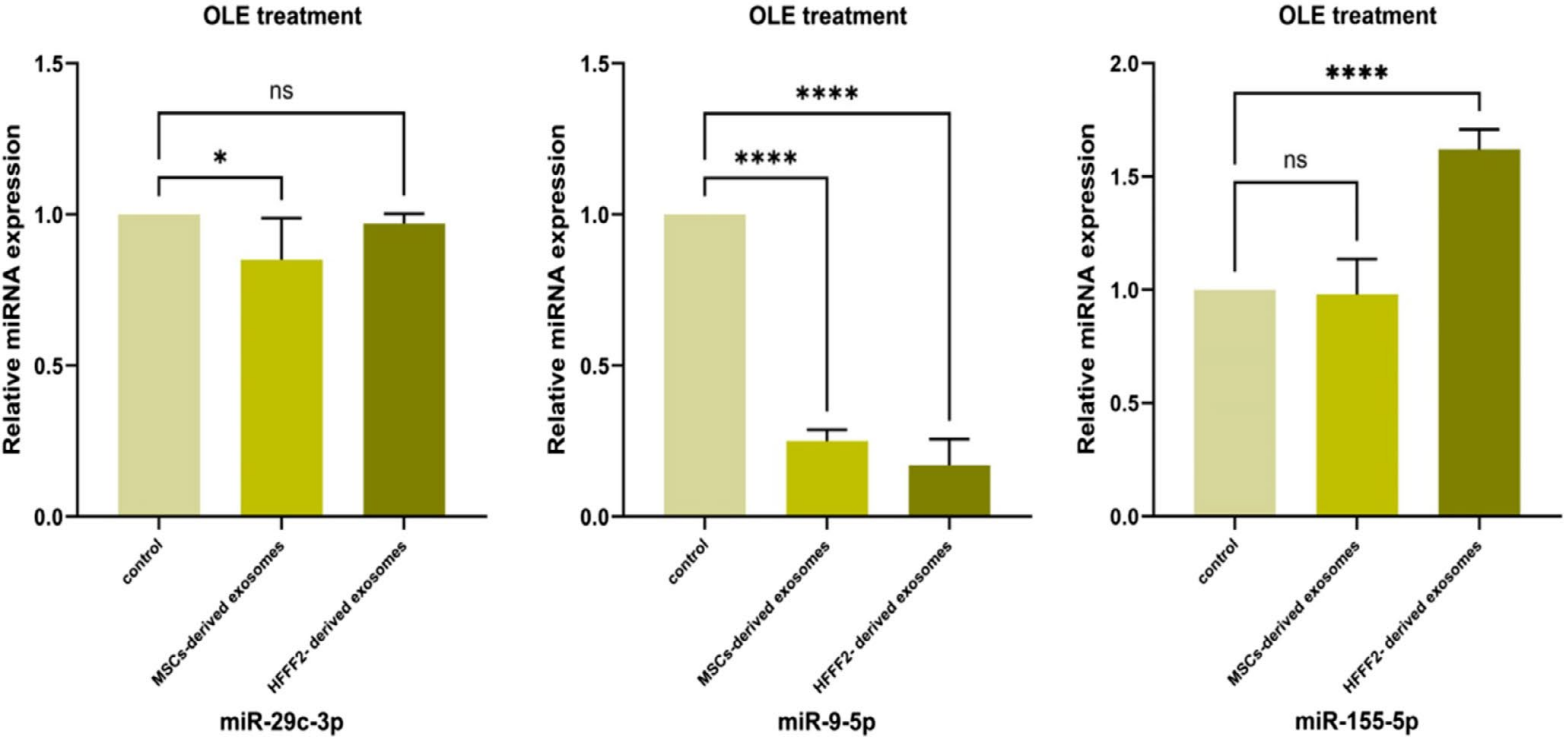
Relative expression of miRNAs (*miR‐29c‐3p, miR‐9‐5p*, *miR‐155‐5p*) in manipulated (OLE‐treated) exosomes, (ns) nonsignificant, (*) *p* ≤ 0.05, (**) *p* ≤ 0.01, (*** or more) *p* ≤ 0.001.

All of the examined miRNA expression was analyzed in exosomes derived from MSCs and HFFF2 OLE‐treated. The results revealed a significant (*p* ≤ 0.05) decrease in the relative expression of *miR‐29c‐3p*, and *miR‐9‐5p* in both exosomes derived from MSCs and HFFF2 cells OLE‐treated (Figure 6). But this decrease in exosomes derived from MSCs was not significant for *miR‐155‐5p* expression. On the other hand, the exosomes derived from HFFF2 under OLE treatment showed a significant (*p* ≤ 0.05) increase (Figure 6).

#### 3.4.4 The miRNAs Expression in HFFF2 Cell Line Treated With Manipulated Exosomes

**FIGURE 7:**
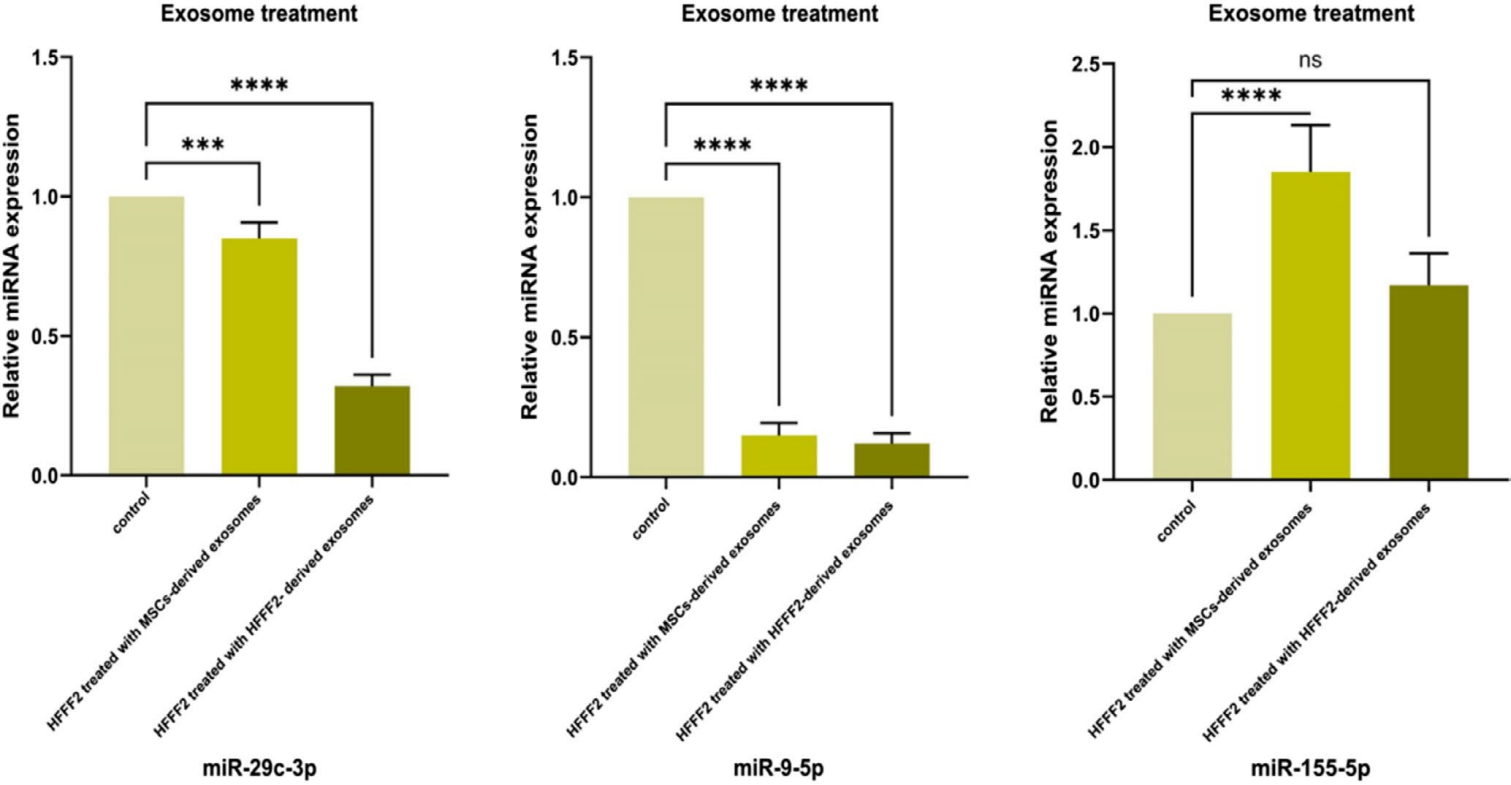
Relative expression of miRNAs (*miR‐29c‐3p, miR‐9‐5p*, *miR‐155‐5p*) in HFFF2 cells treated manipulated (OLE‐treated) exosomes, (ns) nonsignificant, (*) *p* ≤ 0.05, (**) *p* ≤ 0.01, (*** or more) *p* ≤ 0.001.

The studied miRNA expression was evaluated in HFFF2 cells treated with exosomes derived from MSCs and HFFF2 OLE‐treated. A significant (*p* ≤ 0.05) reduce in the relative expression of *miR‐9‐5p* was seen in both treatments in HFFF2 cells (Figure 7). In the case of *miR‐29c‐3p* relative expression, we observed a significant (*p* ≤ 0.05) decrease only when treated with exosomes derived from MSCs OLE‐treated. But this downregulation in HFFF2 cells treated with exosomes derived from HFFF2 OLE‐treated was not significant for *miR‐29c‐3p* and also *miR‐155‐5p* expression. Also, the HFFF2 cells treated with exosomes derived from MSCs OLE‐treated showed a significant (*p* ≤ 0.05) increase (Figure 7).

## 4 Discussion

**FIGURE 8:**
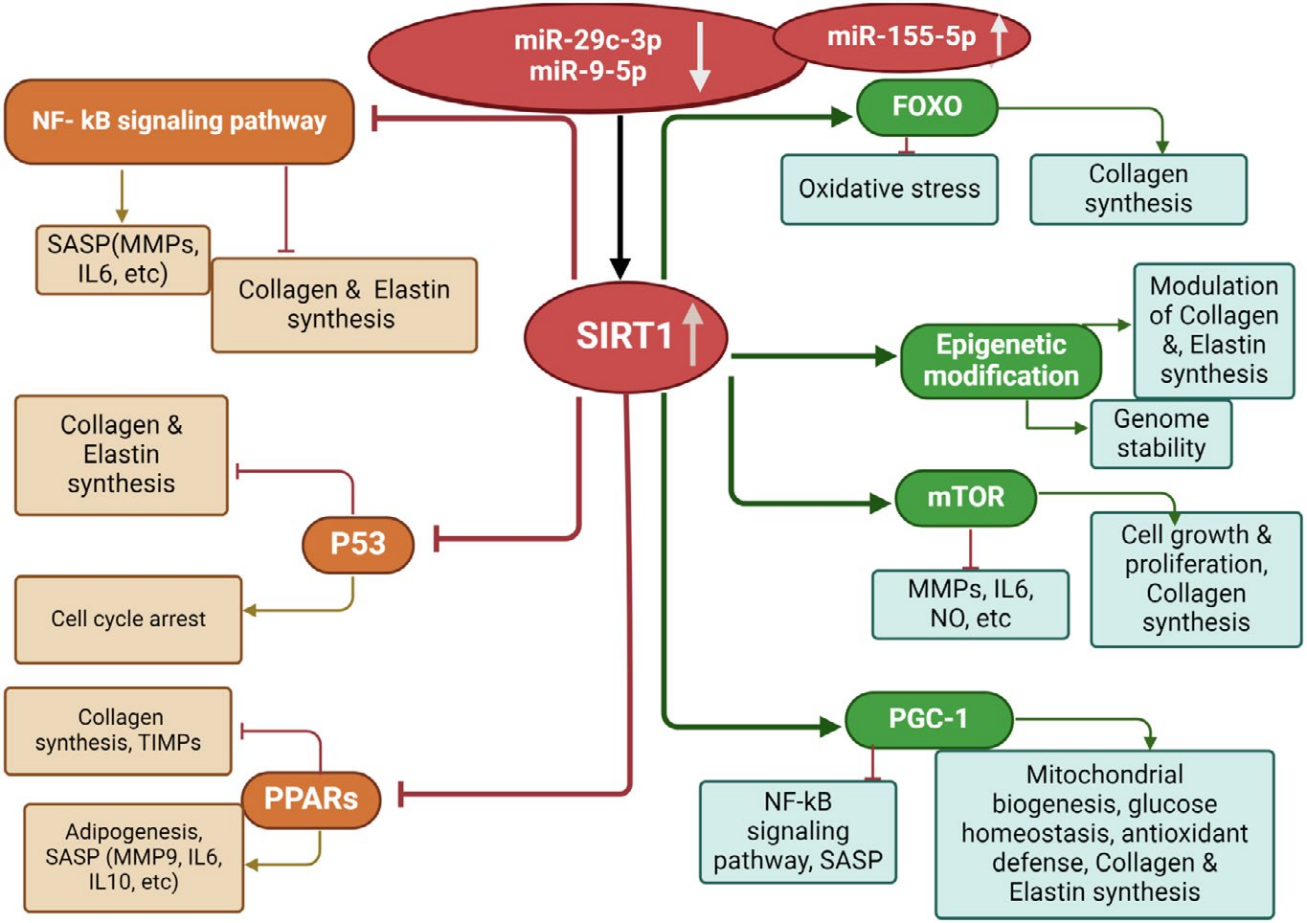
SIRT1 and skin aging related pathways.

According to research, the increase in *SIRT1* gene expression in both types of MSCs and HFFF2 cells under OLE treatment (Figure 4), on the one hand, inhibits inflammation, cell cycle arrest, adipogenesis, and senescence‐associated secretory phenotypes (SASP: *MMPs*, *IL6*, *IL10*, etc.); on the other hand, it increases the inhibition of cell responses to oxidative stress, DNA repair against stress, UV, genomic stability, *MMPs*, and the induction of cell growth, cell proliferation, mitochondrial biogenesis, and antioxidant defense. Generally, inhibiting and inducing these pathways leads to tissue inhibitors of metalloproteinases (TIMPs), *Collagen*, and *Elastin* synthesis (Figure 8). *SIRT1* helps to maintain overall skin health by negatively regulating *MMPs*, helping to maintain the integrity of the ECM and protecting against damage caused by UV radiation and other factors (Bielach‐Bazyluk et al. 2021; Choi et al. 2020; Oh et al. 2023; Orioli and Dellambra 2018; Sung et al. 2024; Vidović and Ewald 2022). So, treatments aiming at increasing *SIRT1* activity or expression may demonstrate great potential for reducing skin aging. OLE therapy has demonstrated the ability to improve the lifespan of normal human fibroblasts by enhancing proteasome activity (Micheli et al. 2023). OLE is seen as a promising agent for the prevention of age‐related diseases, and its influence on *SIRT1* and associated pathways could increase skin health (Micheli et al. 2023).

Treatment of exosomes derived from MSCs and HFFF2 cells OLE‐treated showed that the *SIRT1* gene increased significantly under the influence of exosomes derived from MSCs and HFFF2 cells (Figure 5). The result of OLE treatment was consistent with the result of exosomes derived from cells under OLE treatment. The cause of this result can be attributed to the cell matrix components containing OLE and the influence of OLE on the expression of miRNAs found in exosomes derived from OLE‐treated cells. Studies revealed that treatments applied to cells can greatly change the miRNA content of secreted exosomes, which in turn influences the phenotype of recipient cells. This finding opens new opportunities for therapeutic interventions and emphasizes the need of considering exosomal miRNA changes when developing cell‐based treatments (Nedaeinia et al. 2024).

We also evaluated the role of OLE in the content of exosomal miRNAs derived from MSCs and HFFF2 cells. The findings showed that OLE in the exosomes of both types of cells generally upregulated the expression of *miR‐155‐5p* (Figure 6) and downregulated the expression of *miR‐29c‐3p*, and *miR‐9‐5p* (Figure 6). The downregulation in *miR‐29c‐3p* (Wang et al. 2021), and *miR‐9‐5p* (Wu et al. 2023) expression can be explained by the upregulation in *SIRT1* gene expression. These changes have a direct effect on the *SIRT1* gene. Therefore, one of the mechanisms of *SIRT1* gene expression change can be through the effect of OLE on the miRNAs.

The expression of studied miRNAs in fibroblast cells (HFFF2) was analyzed under the influence of exosomes derived from MSCs and HFFF2 OLE‐treated. The results indicated a decrease in the expression of miRNAs *miR‐29c‐3p*, and *miR‐9‐5p*. This reduction follows our expectations, as these miRNAs target the *SIRT1* gene. However, it confirms the significant interaction of miRNAs, including *miR‐29c‐3p*, and *miR‐9‐5p* with the *SIRT1* gene (Wang et al. 2021; Wu et al. 2023; Xie et al. 2023; Zeng et al. 2020). In our study, both *miR‐155‐5p* and *SIRT1* were found to be upregulated, which may seem paradoxical considering their known pro‐inflammatory roles in aging and inflammation. However, recent evidence suggests a more intricate relationship between these molecules. Specifically, *miR‐155‐5p* directly interacts with *SIRT1*, a crucial regulator of cellular aging and inflammation, influencing cell survival and stress responses. The miR‐155‐5p/SIRT1/TLR4 signaling pathway regulates inflammation and oxidative stress, and its modulation can protect cells from damage and inflammation (e.g., *Ginkgo biloba* extract reduces harm through this pathway) (Liu et al. 2025). In certain cases, the overexpression of *miR‐155‐5p* and *SIRT1* may diminish pro‐inflammatory signals and promote cellular repair and longevity in response to stress. Our data suggest that the co‐upregulation of *miR‐155‐5p* and *SIRT1* may enhance cellular homeostasis and reduce aging‐related damage, rather than opposing the observed anti‐aging effects. The elevated expression of *miR‐155‐5p* in our study may not counteract anti‐aging effects but could be integral to a complex regulatory network involving *SIRT1* that enhances skin cell resilience and longevity, aligning with prior molecular findings (Liu et al. 2025; Wang et al. 2018). The dynamics of this interaction in skin aging models require additional investigation.

In general, manipulated (OLE‐treated) exosomes have shown similar effects on *SIRT1* gene expression as direct OLE treatment, justifying that the increase in *SIRT1* gene expression occurs via the altered expression of miRNAs (*miR‐29c‐3p*, *miR‐9‐5p*). Exosomes effectively transfer OLE and its molecular effects on cells (mRNA, miRNA, DNA, protein, etc.) without triggering the immune system and, facilitating OLE entry, suggesting they are a more effective therapeutic approach than using OLE alone.

On the other hand, the present study is limited by its exclusive reliance on in vitro HFFF2 cell models, which, although informative, fail to fully replicate the complexities of in vivo skin aging processes. Future research should utilize mouse skin aging models to systematically evaluate the anti‐aging efficacy and safety profile of OLE‐treated exosomes, as suggested in recent literature. In this study, although western blotting and dynamic light scattering (DLS) were used in this study to characterize the exosomes, we recognize that these techniques, despite their widespread use, only offer a limited amount of information about the precise morphology and ultrastructure of exosomes. As a gold standard for exosome validation, transmission electron microscopy (TEM) provides high‐resolution visualization of vesicle size, shape, and membrane integrity all of which are essential for verifying the identity and purity of exosomes. Recent guidelines and studies underscore the necessity of integrating biochemical (e.g., western blot for exosomal markers), biophysical (e.g., dynamic light scattering or nanoparticle tracking analysis), and morphological (e.g., transmission electron microscopy) methodologies for thorough exosome characterization. The lack of TEM investigation in our current study constitutes a restriction, as it prevents direct morphological evaluation of the isolated vesicles (Imanbekova et al. 2022; Moghassemi et al. 2024). Also, recent studies have highlighted the significance of accurate measurement of bioactive chemicals in exosomes to assess their loading efficiency and therapeutic potential (Chen et al. 2024). Subsequent research will utilize advanced analytical methods, including high‐performance liquid chromatography (HPLC) and mass spectrometry, to measure the OLE concentration in exosome preparations. This will enable us to gain a deeper insight into the pharmacokinetics, loading accuracy, and delivery efficacy of OLE‐loaded exosomes. Overcoming this limitation is essential for improving our exosome‐based delivery system and increasing the clinical significance of OLE in anti‐aging therapies, aligning with contemporary developments in exosome engineering and standardization (Chen et al. 2024; Villarreal‐Gómez et al. 2025).

## 5 Conclusion

The results for OLE and manipulated (OLE‐treated) exosomes treatments showed a similar effect on *SIRT1* gene expression, which justified the increase in *SIRT1* gene expression by altering the expression of *miR‐29c‐3p* and *miR‐9‐5p*. The correlation between the expression of miRNAs in HFFF2 cells and manipulated (OLE‐treated) exosomes suggests that treatment conditions influence the contents of intracellular miRNAs. The use of manipulated (OLE‐treated) exosomes appear to be more effective in enhancing *SIRT1* gene expression in skin fibroblast cells (HFFF2) because exosomes transfer OLE to cells without triggering the immune system, and they facilitate OLE entry into cells, making it a more effective therapeutic approach than using OLE alone. The selective absorption mechanism of OLE via glucose transporters limits its absorption. Therefore, manipulated (OLE‐treated ≃ 400 μg/mL) exosomes decreased the expression of *miR‐29c‐3p* and *miR‐9‐5p* miRNAs and caused *SIRT1* overexpression as a result of exosome treatment at 50 μg/mL (protein concentration of exosomes) in fibroblast cells in vitro. Exosome therapy is a revolutionary facial rejuvenation method that utilizes cellular communication. Proposing to conduct clinical trials could enhance the findings and extend the research.

## Author Contributions

**Naeimeh Safavizadeh:** conceptualization (equal), formal analysis (equal), funding acquisition (equal), investigation (equal), methodology (equal), resources (equal), software (equal), visualization (equal), writing – original draft (equal). **Zahra Noormohammadi:** project administration (equal), resources (equal), supervision (equal), validation (equal), writing – review and editing (equal). **Mohammad Zaefizadeh:** investigation (equal), methodology (equal), project administration (equal), software (equal), supervision (equal), validation (equal), writing – review and editing (equal). **Kazem Nejati Koshki:** project administration (equal), supervision (equal), validation (equal), writing – review and editing (equal).

## Ethics Statement

This study was performed in accordance with the Declaration of Helsinki, and the protocol received approval from the Research Ethics Committee of Azad University, Ardabil Branch, Iran, with ethics code IR.IAU.ARDABIL.REC.1402.054.

## Conflicts of Interest

The authors declare no conflicts of interest.

## Data Availability

All data generated or analyzed during this study are included in this published article. For any further clarification or additional information, the corresponding author is available upon request.
